# Supplementary material for: Concerted EP2 and EP4 Receptor Signaling Stimulates Autocrine Prostaglandin E2 Activation in Human Podocytes
Source: Cells. 2020 May 19;9(5):1256. doi: 10.3390/cells9051256 (PMC7290667; doi:10.3390/cells9051256)
Supplement: Supplementary file 1 [file cells-09-01256-s001.pdf]

Supplemental Material

S 1. Immunofluorescence of Synaptopodin, Podocin, and Nephrin

Differentiated phenotype of hPC was confirmed by analysis of the marker synaptopodin by immunofluorescence. Briefly, cells were seeded on 8-chamber-glass slides at 12 x 10^3 cells per well (NUNC Lab-Tek II Chamber Slide, cat. no. 154534) and kept in RPMI-1640 medium with supplements for adherence overnight. Medium was then discarded and cells were washed twice with PBS. Fixation of cells was achieved with 80% acetone/PBS at 4 °C for 10 min. Afterwards, cells were washed three times with cold PBS. Blocking solution consisting of PBS+5%FBS was applied for 20 min at room temperature. Primary antibody mouse anti-synaptopodin (cat. no. 65194, Progen Biotechnik GmbH, Germany) diluted 1:25 was added and incubation lasted 1 h at room temperature. After three washing steps, the secondary antibody donkey anti-mouse IgG Alexa Fluor Plus 594 (cat. no. A32744, Thermo Fisher Scientific, Germany) diluted 1:200 was added and incubated for 1 h at room temperature in darkness. Cells were washed twice with PBS and nuclei were stained with 4′,6-diamidino-2-phenylindole (DAPI, cat. no. P36931, Invitrogen, Germany) for 24 h at room temperature in darkness. Immunofluorescence was detected at 405 nm (DAPI) and 594 nm (donkey anti mouse for synaptopodin) on a Leica confocal SPE microscope (Figure S1 (a) and (b)).

Additionally, immunofluorescence for the podocyte markers podocin and nephrin was performed in 24-well plates. For nephrin, the protocol of synaptopodin staining was followed using rabbit anti-nephrin (cat. no. PA5-72826, Invitrogen, Germany) diluted 1:50 as primary antibody and goat anti-rabbit Alexa Fluor Plus 488 (cat. no. A32732, Invitrogen, Germany) 1:200 as secondary antibody. Nuclei staining was performed using DAPI 1:3000 (cat. no. D1306, Thermo Fisher, Germany) with incubation for 10 min at room temperature in darkness. Cells were finally washed twice with PBS and covered with Fluoromount (cat. no. 00-4958-02, Invitrogen, Germany). For podocin, fixation of cells was achieved by 4% paraformaldehyde+1 mM $MgCl_2$+0.5% Triton X100 using 800 µL per well and an incubation time of 10 min at room temperature. After three washing steps with PBS, blocking solution consisting of PBS+5% FBS+0.1% Triton X100 was added to each well and incubated for 20 min at room temperature. Primary antibody rabbit anti-podocin (cat. no. P0372, Sigma-Aldrich, Germany) diluted 1:50 was added and incubation lasted 1 h at room temperature. Afterwards, the same protocol was followed as for podocin staining. Immunofluorescence was detected at 405 nm (DAPI) and 488 nm (goat anti-rabbit for podocin and nephrin) on an EVOS fluorescence microscope (Thermo Fisher, Germany) (Figure S1 (c)-(e)).

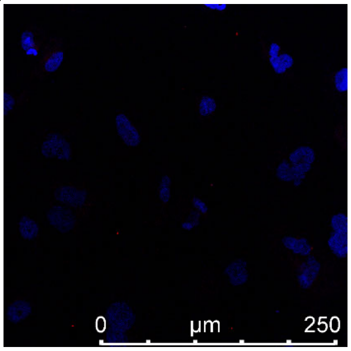

(**a**)

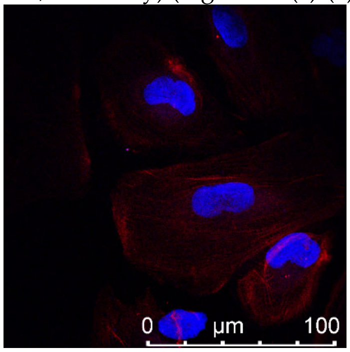

(**b**)

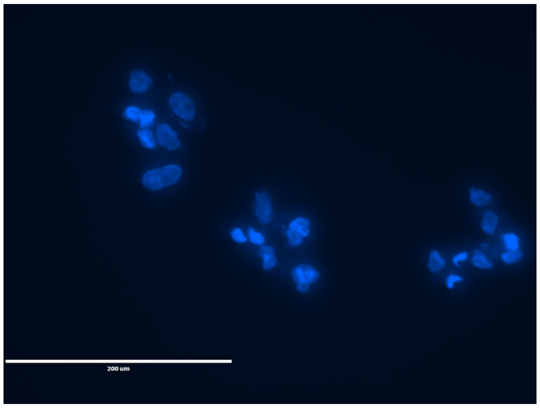

(**c**)

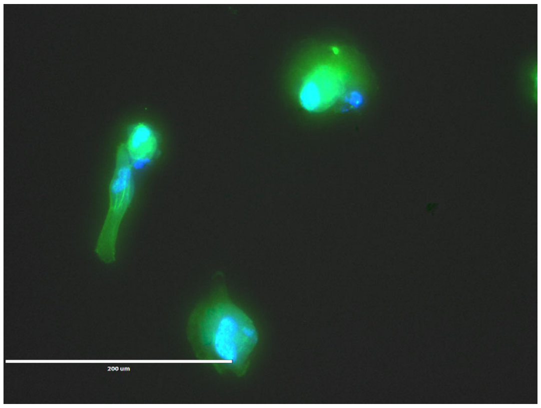

(**d**)

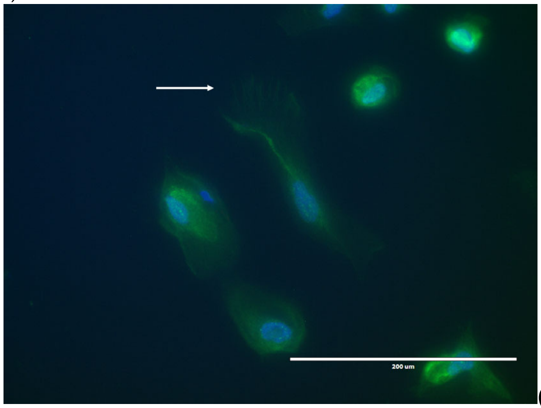

(**e**)

Figure S1. Differentiation of hPC was confirmed by immunofluorescence of synaptopodin, podocin, and nephrin. (**a**) Control without primary antibody revealed no unspecific binding of secondary antibody donkey anti-mouse IgG Alexa Fluor Plus 594, overlay with DAPI indicated nuclei. Scale bar 250 µm; (**b**) Synaptopodin (red) was expressed by differentiated hPC. Nuclei staining was performed with DAPI. Scale bar 100 µm; (**c**) Control without primary antibody revealed no unspecific binding of secondary antibody goat anti-rabbit Alexa Fluor Plus 488, overlay with DAPI indicated nuclei. Scale bar 200 µm; (**d**) Podocin (green) and (**e**) nephrin (green) were expressed by differentiated hPC. Foot processes are visible in (**e**) indicated by white arrow. Scale bars 200 µm.

S 2. Additional Characterization of hPC

Cellular appearance differed between differentiated hPC exhibiting an arborized shape (Figure S2a), and proliferating, undifferentiated hPC that showed typical "cobblestone" pattern (Figure S2b). This appearance was also described before [1]. Furthermore, synaptopodin (*SYNPO*) gene expression (forward primer (5′-3′) – gaggacctagcagacgttgg, reverse primer (5′-3′) – tctgagtacccctccatgct) was shown on differentiated hPC, while undifferentiated hPC did not express *SYNPO* mRNA (Figure S2c). Additionally, nephrin and podocin protein were detected by western blot using rabbit anti-nephrin antibody (cat. no. PA5-72826, Thermo Scientific) and rabbit anti-podocin antibody (cat. no. P0372, Sigma), both diluted 1:500 and incubated at 4 °C overnight as primary antibodies and horseradish peroxidase conjugated goat anti-rabbit (cat. no. sc-2004, Santa Cruz Biotechnology) as secondary antibody. Nephrin and podocin were expressed on differentiated and proliferating podocytes, albeit the undifferentiated hPC exhibited weaker expression of both (Figure S2d, e).

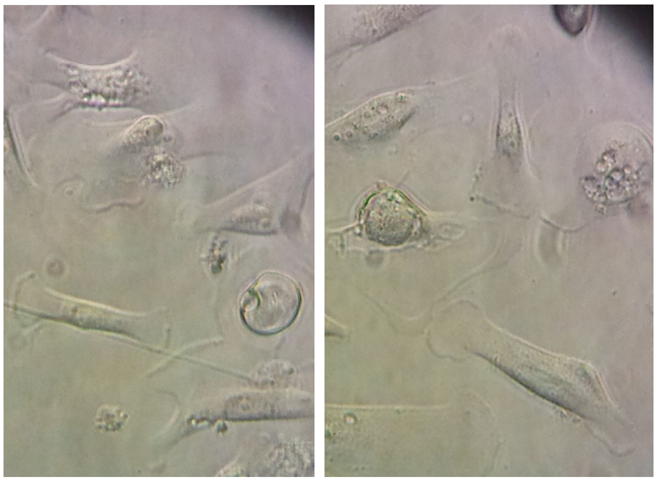

(**a**)

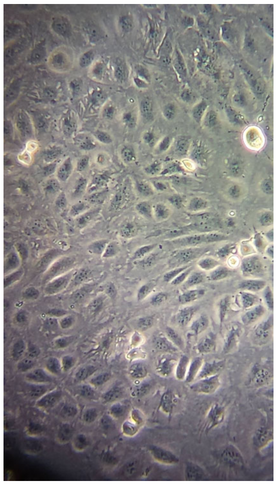

(**b**)

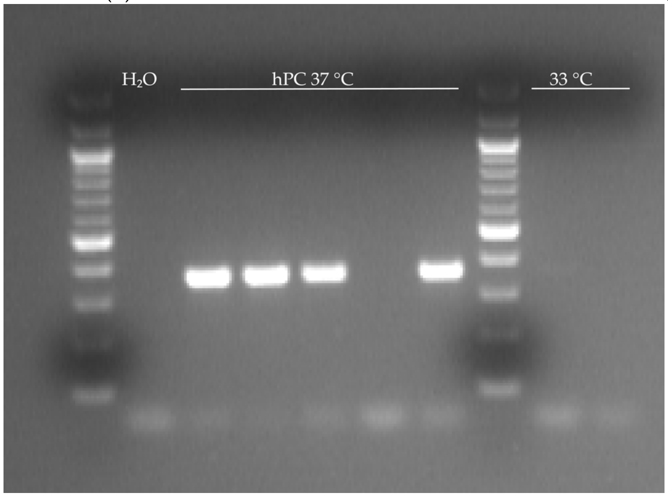

(**c**)

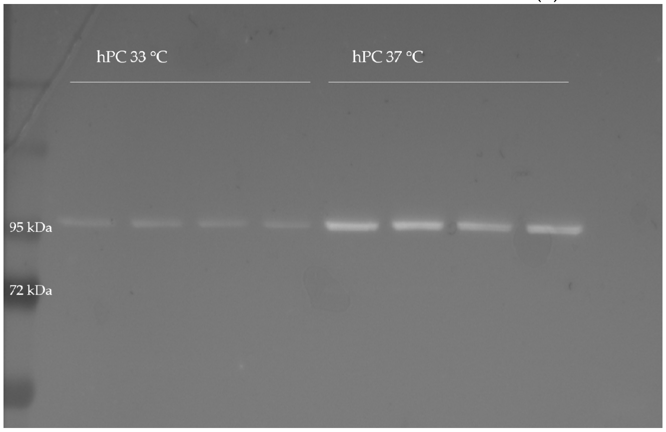

(**d**)

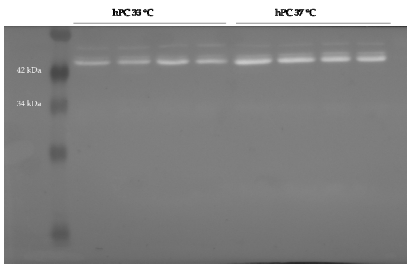

(**e**)

Figure S2. Characterization of differentiated and undifferentiated hPC. Light microscopy revealed an arborized shape of differentiated hPC at 37 °C (**a**), and a cobblestone pattern of undifferentiated hPC at 33 °C (**b**); (**c**) PCR revealed *SYNPO* gene expression in differentiated hPC (“hPC 37 °C”) and no expression in undifferentiated hPC (“33 °C”); (**d**) nephrin and podocin (**e**) protein were detected by western blot in undifferentiated (“hPC 33 °C”) and differentiated (“hPC 37 °C”) hPC.

S 3. Expression of EP in hPC

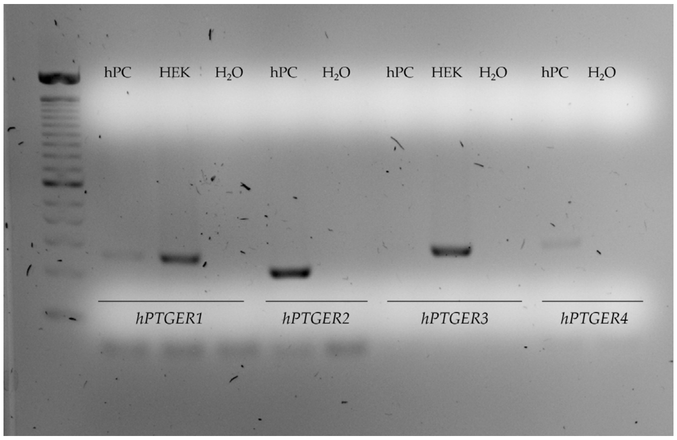

Figure S3. Characterization of hPC for EP receptor expression. *PTGER1* (EP1), *PTGER2* (EP2), and *PTGER4* (EP4) mRNA is present in differentiated hPC. HEK293 (HEK) served as a positive control where indicated.

S 4. COX2 expression in hPC after co-incubation with PGE2 1 µM and EP2 antagonist 1 µM

In a pilot test, we analyzed whether the selective EP2 antagonist PF-04418948 (1 µM) could abolish the effects of 1 µM PGE_2 on *COX2* mRNA expression. Stimulation of hPC with PGE_2 1 µM for 2 h revealed an increase in *COX2* mRNA (Figure S4). Upon PGE_2 stimulation, co-incubation with 1 µM of the selective EP2 antagonist PF-04418948 did not inhibit the PGE_2-mediated increase in *COX2* mRNA (Figure S4).

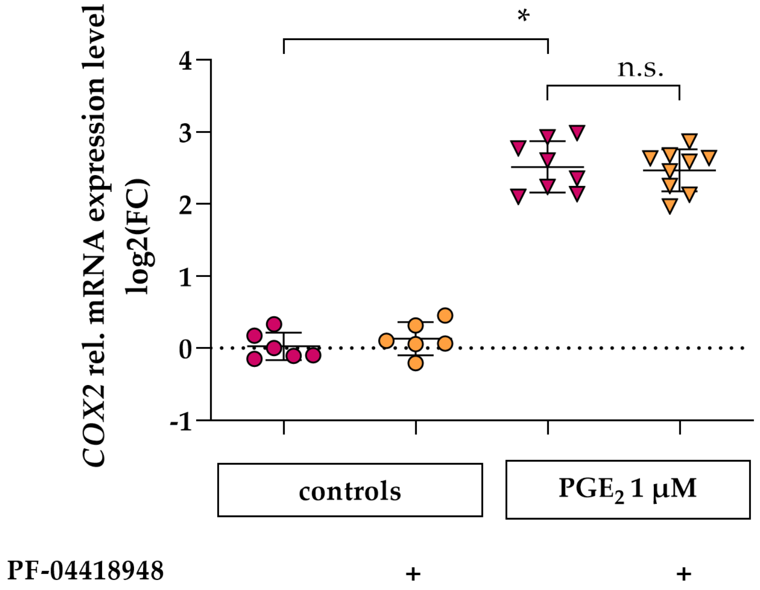

Figure S4. EP2 antagonist does not inhibit PGE_2 mediated *COX2* upregulation when applied with the same concentration as PGE_2. *COX2* mRNA-levels following PGE_2 stimulation with 1 µM for 2 h without concomitant EP2 antagonist (pink triangles) compared to controls without PGE_2 (pink circles), after co-incubation with 1 µM EP2 antagonist (PF-04418948, orange triangles) compared to controls without PGE_2 (orange circles). Each datapoint represents a single sample and plotted as mean ± SD (horizontal lines) per treatment group. Each treatment group consisted of n=6–9 replicates analyzed in a single experiment. Statistics: *, $p<0.01$; n.s., not significant, assessed by two-tailed Student's t-test.

S 5. LC/ESI-MS/MS for Analysis of Prostaglandins

Table S1. Multiple Reaction Monitoring in negative mode for LC/ESI-MS/MS.

Compound Name	Precursor Ion	Product Ion	Mass Res	CE (V)	Ret Time (min)
Tetranor PGFM	329	249	Unit	26	3
Tetranor PGFM	329	239	Unit	25	3
Tetranor PGFM	329	149	Unit	31	3
Tetranor PGFM	329	129	Unit	33	3
6-keto-PGF1a	369.2	245	Wide	28	4.61
6-keto-PGF1a	369.2	207	Wide	22	4.61
6-keto-PGF1a	369.2	163	Wide	29	4.61
TXB2-1	369.2	195.1	Wide	12	7.04
TXB2-1	369.2	169.1	Wide	14	7.04
11ß-PGF2a	353.2	273.2	Wide	22	7.3
11ß-PGF2a	353.2	193.1	Wide	26	7.3
11ß-PGF2a	353.2	291.2	Wide	21	7.3
PGF2a	353.2	291	Wide	23	8.84
PGF2a	353.2	273	Wide	22	8.84
PGF2a	353.2	193	Wide	28	8.84
PGF2a	353.2	165	Wide	27	8.84
PGE2-D4	355.2	275.2	Wide	18	9.84
PGE2	351.2	315.2	Wide	10	9.94
PGE2	351.2	271.2	Wide	18	9.94
PGE2	351.2	189.1	Wide	20	9.94
PGF2a-15-keto	351.2	315	Wide	11	10.64
PGF2a-15-keto	351.2	191	Wide	28	10.64
PGD2	351.2	315.2	Wide	11	11.3
PGD2	351.2	271.2	Wide	18	11.3
PGD2	351.2	233.2	Wide	10	11.3
PGD2	351.2	189.1	Wide	20	11.3
PGE2-15-keto	349.2	287.2	Wide	14	11.8
PGE2-15-keto	349.2	235.2	Wide	14	11.8
PGE2-15-keto	349.2	113.1	Wide	19	11.8
PGF2a-13,14dihydro-15keto	353.2	291	Wide	22	13.7
PGF2a-13,14dihydro-15keto	353.2	183	Wide	29	13.7
PGF2a-13,14dihydro-15keto	353.2	113	Wide	29	13.7
PGE2-13,14-dihydro-15-keto	351.2	315.2	Wide	20	14.4
PGE2-13,14-dihydro-15-keto	351.2	235.2	Wide	22	14.4
PGE2-13,14-dihydro-15-keto	351.2	175.2	Wide	22	14.4
PGE2-13,14-dihydro-15-keto	351.2	113.1	Wide	28	14.4
PGD2-13,14-dihydro-15-keto	351.2	315.2	Wide	12	17.7
PGD2-13,14-dihydro-15-keto	351.2	207.1	Wide	20	17.7
PGD2-13,14-dihydro-15-keto	351.2	175.2	Wide	20	17.7
PGD2-13,14-dihydro-15-keto	351.2	163.1	Wide	26	17.7
PGJ2	333.2	271.2	Wide	16	20.75
PGJ2	333.2	233.1	Wide	9	20.75
PGJ2	333.2	189.1	Wide	17	20.75
PGJ2-delta12	333.2	271.2	Wide	16	21.1
PGJ2-delta12	333.2	233.1	Wide	9	21.1
PGJ2-delta12	333.2	189.1	Wide	17	21.1
PGJ2-15-deoxy-delta 12,14	315.2	271.2	Wide	12	26.2
PGJ2-15-deoxy-delta 12,14	315.2	243.2	Wide	20	26.2
PGJ2-15-deoxy-delta 12,14	315.2	217.1	Wide	18	26.2

Compound Name	Precursor Ion	Product Ion	Mass Res	CE (V)	Ret Time (min)
PGJ2-15-deoxy-delta 12,14	315.2	203.1	Wide	24	26.2
PGJ2-15-deoxy-delta 12,14	315.2	158.2	Wide	20	26.2

1. Saleem, M.A.; O'Hare, M.J.; Reiser, J.; Coward, R.J.; Inward, C.D.; Farren, T.; Xing, C.Y.; Ni, L.; Mathieson, P.W.; Mundel, P. A conditionally immortalized human podocyte cell line demonstrating nephrin and podocin expression. *Journal of the American Society of Nephrology : JASN* **2002**, *13*, 630-638.
